# Numerical Modeling in Membrane Processes

**DOI:** 10.3390/membranes12111030

**Published:** 2022-10-23

**Authors:** Sébastien Déon, Patrick Dutournié

**Affiliations:** 1 Institut UTINAM (UMR CNRS 6213), Université de Bourgogne Franche-Comté, 16 Route de Gray, CEDEX, 25030 Besançon, France; 2 Institut de Science des Matériaux de Mulhouse (IS2M-UMR CNRS 7361), Université de Haute-Alsace, 3Bis Rue Alfred Werner, CEDEX, 68098 Mulhouse, France

Membrane processes have demonstrated their enormous potential for water treatment, either by removing organic and mineral contaminants before permeating stream discharge, or by concentrating high added-value compounds in retentate stream. Although the advantages and drawbacks of the various membrane processes are well known, the mechanisms governing their filtration performances are usually not fully understood, and discussion is often still open. For this purpose, researchers have been developing numerical models for decades to describe the transport of species through membranes and predict their performances for specific applications. Numerical modeling can be useful in many aspects of membrane science and can help to solve many scientific issues. A numerical approach can be used to model the physical mechanisms governing fluid flow or mass transfer, and then applied to various membrane processes, such as pressure-driven, concentration-driven, electrically driven, or thermally driven processes.

This Special Issue on “Numerical Modeling in Membrane Processes” provides examples of original works or reviews of the literature dealing with the use of mathematical models to understand, describe, predict, or optimize the performances of membrane processes. 

Quezada et al. [1] reviewed the various phenomenological and non-phenomenological models for the prediction of permeate flux in Ultrafiltration (UF), as well as a comparison of their predictive ability. Selected models were tested with data for three fruit juices (bergamot, kiwi, and pomegranate) filtered by a cross-flow system over the course of 10 h. The robust statistical examination, including a residual analysis, suggests that non-phenomenological models are a useful tool from a practical point of view, whereas phenomenological models are more suitable for scaling-up and for understanding the UF process. 

Kim et al. [2] numerically investigated the fouling mitigation using chaotic advection caused by herringbone-shaped grooves in a flat membrane module. In their work, they showed that under the optimum groove geometry, foulants near the membrane are trans-ported back to the bulk flow via the downwelling flows, distributed uniformly in the entire channel via chaotic advection. 

Park et al. [3] studied the impact of membrane geometry on the transport of colloidal particles in inside-out crossflow ultrafiltration. They have, for instance, highlighted that, irrespective of many input parameters characterizing an UF experiment and its mem-brane geometry, the process indicators are determined by three independent dimension-less variables only. 

Chae et al. [4] formulated in their work a mathematical relation between the driving pressures of membrane-based desalting processes, which takes into consideration the energy loss for each driving pressure. This study diagnosed the commercial advantage of Reverse Osmosis (RO) over Forward Osmosis (FO)/Pressure-Retarted Osmosis (PRO) and suggested optimization sequences applicable to each process. 

Choi et al. [5] analyzed the available energy (exergy) in a Direct Contact Membrane Distillation (DCMD) system, using computational fluid dynamics (CFD) to investigate the hydrodynamic and thermal conditions in the module. 

Their study revealed that exergy destruction in the permeate occurred near the feed inlet, and the effect became less influential closer to the feed outlet. Their analysis of exergy flows also showed that the efficiency loss in the permeate side corresponded to 32.9–45.3% of total exergy destruction. 

Gu et al. [6] implemented CFD simulations to establish new, dimensionless correlations for the concentration polarization (CP) modulus and friction factor in Spacer-Filled Reverse Osmosis Membrane Modules. The authors concluded that their correlations for the CP modulus has the advantage of being directly usable to estimate the impact of permeate flux on concentration polarization at a membrane surface, without resorting to the film theory. 

Xie et al. [7] coupled CFD modeling with solute transport evaluation in order to study hydrodynamics and concentration polarization in Forward Osmosis (FO) and Pressure-Retarted Osmosis (PRO). Simulations showed that concentration and velocity profiles are impacted by spacers, which could reduce or enhance water flux depending on the inlet flow velocity and the distance between membrane and spacer. 

Zhang et al. [8] simulated the continuous extraction of lithium ions from diluted salt lake brines with high Mg^2+^/Li^+^ ratio based on free flow ion concentration polarization in a microfluidic system. In their study, the authors numerically showed that this method is able to decrease the Mg^2+^/Li^+^ ratio significantly, and has great potential as preprocessing technology for lithium extraction from salt lake brines. 

Zhu et al. [9] used a steric, electrostatic, and dielectric mass transfer model to investigate the separation mechanisms of typical antibiotic sulfadiazine by various nanofiltration (NF) membranes. The authors showed that sulfadiazine rejection and membrane sequence obtained by the model are qualitatively consistent with experiments. 

Dutournié et al. [10] proposed a novel numerical procedure based on physical simplifications, which allows the estimation of a range of values for the dielectric constant of the confined solution and membrane charge density required to model the transport through NF membranes. It is shown in this study that the evolution of the interval of membrane charge with salt concentration can be described by the Langmuir–Freundlich hybrid adsorption isotherm, which allowed a good prediction ability, irrespective of the salt and membrane considered. 

Nagy et al. [11] compared the linear Van’t Hoff approach and the real osmotic pressure values obtained using OLI Stream Analyzer for various dilutions of NaCl solutions. Their results indicated that the disparity in the predicted osmotic pressure assessed with the two methods can reach 30%, depending on the solute concentration, while that in the predicted power density can exceed 50%. Hence, the difference in structural parameter values predicted by the two methods is also significant, and can even exceed the 50–70% range, depending on the operating conditions. 

Jokic et al. [12] examined a non-recurrent feed-forward Artificial Neural Network (ANN) with one hidden layer to model microfiltration (MF) of Bacillus velezensis cultivation broth. For MF experiments, Kenics static mixer and two-phase flow were investigated, either alone or in combination, to improve permeate flux. This study confirmed the predictive ability of the ANN model for the estimation of permeate flux. The optimal ANN topology was 5-13-1, trained by the Levenberg–Marquardt algorithm and with hyperbolic sigmoid transfer function between the input and the hidden layer. 

Skolotneva et al. [13] proposed a 1D convection–diffusion–reaction model to describe the transport and oxidation of oxalic acid and oxygen evolution in the flow-through electrochemical oxidation system, considering Reactive Electrochemical Membrane (REM). Their model provided an understanding of the process and allowed the estimation of concentration, current density, potential, and overpotential distributions in REM. They also highlighted that the oxygen evolution reaction notably affects the process, even if its contribution decreases with increasing total organic carbon flux. 

Lukitsch et al. [14] conducted CFD simulations to understand the remaining difference between the CO_2_ removal rate which was determined in vivo with porcine blood from that determined in vitro with water. This study indicated that the main CO_2_ transport resistance behaves generally differently in blood and water. The authors concluded their work by mentioning that studies of the CO_2_ boundary layer should be preferably conducted with blood, whereas water tests should be favored for the determination of total CO_2_ removal performance of oxygenators. 

Wu et al. [15] performed a 3D numerical simulation in order to investigate the barrier property of mixed-matrix membranes (MMMs) and their effective membrane gas permeability. The authors highlighted that horizontally-aligned thin cuboid nanoparticles offer superior barrier properties than spherical nanoparticles for an identical solid volume fraction. A novel ANN model based on multivariable regression analysis was developed, and it was able to predict the relative permeability of MMMs over an extensive range of solid volume fraction and aspect ratios. 

Osterroth et al. [16] used a CFD study for the attachment of microcapsules on the membrane surface and its influence on the flow field in a cross-flow membrane module. This study allowed them to conclude that the glued configuration provides a lower transmembrane pressure than the configuration where microcapsules are added during fabrication. 

Finally, Nunes et al. [17] studied the separation of oily water using a new configuration of hydrocyclone, equipped with a porous ceramic membrane in the wall of the conical part (filtering hydrocyclone). An Eulerian–Eulerian approach was used to solve the mass and momentum conservation equations as well as the turbulence model, using the CFD technique. This study demonstrated the high potential of filtering hydrocyclone for the separation of water/oil mixtures. 
